# Healthcare on the brink: navigating the challenges of an aging society in the United States

**DOI:** 10.1038/s41514-024-00148-2

**Published:** 2024-04-06

**Authors:** Charles H. Jones, Mikael Dolsten

**Affiliations:** Pfizer, 66 Hudson Boulevard, New York, New York, 10018 USA

**Keywords:** Geriatrics, Health services, Public health, Epidemiology

## Abstract

The US healthcare system is at a crossroads. With an aging population requiring more care and a strained system facing workforce shortages, capacity issues, and fragmentation, innovative solutions and policy reforms are needed. This paper aims to spark dialogue and collaboration among healthcare stakeholders and inspire action to meet the needs of the aging population. Through a comprehensive analysis of the impact of an aging society, this work highlights the urgency of addressing this issue and the importance of restructuring the healthcare system to be more efficient, equitable, and responsive.

## Introduction

The United States is undergoing a demographic and health transformation that will have profound implications for its healthcare system and society. The population is aging at an unprecedented rate, with the baby boomer generation, defined as those born between 1946 and 1964, reaching retirement age and living longer than ever before. According to the U.S. Census Bureau, by 2030, all baby boomers will be older than 65, leading to about one in every five residents being retirement age^1^. This shift poses a dual challenge for the healthcare system: how to meet the increasing and complex healthcare needs of the elderly, and how to ensure that the system is prepared and equipped to provide quality and equitable care for this growing segment of the population.

The implications of this demographic shift are far-reaching. The healthcare system, as currently structured, is underprepared for the onslaught of demands this aging population will impose^2^. The system is also fragmented, inefficient, and costly, with gaps in coordination, quality, and access. Moreover, the system does not adequately address the social determinants of health, such as income, education, housing, transportation, and social support, that impact the health outcomes and health behaviors of the older adults^3–5^. Nor does it sufficiently engage the older adults and their caregivers in the planning and delivery of care or leverage the potential of community-based and home-based care models, which can improve the access, quality, and affordability of care for the elderly^6^.

However, the challenge does not end there. There is a growing shortage of healthcare providers, which means that the supply and availability of qualified and skilled healthcare professionals, such as physicians, nurses, pharmacists, clinical social workers and technicians, is insufficient and inadequate to meet the demand and need of the population. This phenomenon is even more pronounced in low- and middle-income countries, as well as in the rural and remote areas within the U.S. According to the World Health Organization (WHO), the global health workforce was 43.5 million in 2018, and is projected to grow to 53.9 million by 2030, but still falls short of the estimated demand of 80 million by 2030, resulting in a global shortfall of 18 million health workers, mostly in low- and middle-income countries^7^. In the U.S., a study by the Association of American Medical Colleges (AAMC) predicts a shortage of up to 139,000 physicians by 2033^8^. The shortage of healthcare providers has significant implications for the health sector, as it affects the access, quality, and cost of healthcare, as well as the health outcomes and satisfaction of the population.

As such, the U.S. is facing a healthcare paradox^9^. On one side, there is an aging population with increasing healthcare needs, and on the other, there is a strained healthcare system grappling with workforce shortages, capacity challenges, and fragmentation. Addressing this paradox requires innovative solutions, policy reforms, and a commitment to restructuring the healthcare system to be more efficient, equitable, and responsive to the needs of its aging citizens.

Although many of these issues may be front-of-mind for geriatrics specialists, many other stakeholders in the eldercare system likely do not understand the full scale of challenges brought on by a rapidly aging populace or may underestimate their preparedness for the resulting changes. This was seen in a recent survey of stakeholders in adult vaccine market, which represents a sector critical in the eldercare industry^10^. When presented with the projected rise of adult vaccines, a trend driven largely by the needs of the aging population, stakeholders across the market were unaware of the associated complexities and anticipated minimal challenges in adopting expanded vaccine schedules^10^. While vaccines represent only a portion of the eldercare market, it is reasonable to believe that this lack of a holistic understanding applies to all sectors in the eldercare industry.

This paper aims to stimulate dialogue and collaboration among the healthcare stakeholders, and to inspire action and innovation to address the needs and aspirations of the aging population. To accomplish this, this work will provide a comprehensive and critical analysis of the impact and implications of an aging society to highlight the importance and urgency of this issue.

## Rising tide of healthcare needs: increasing demand and complexity of care

**Fig. 1:**
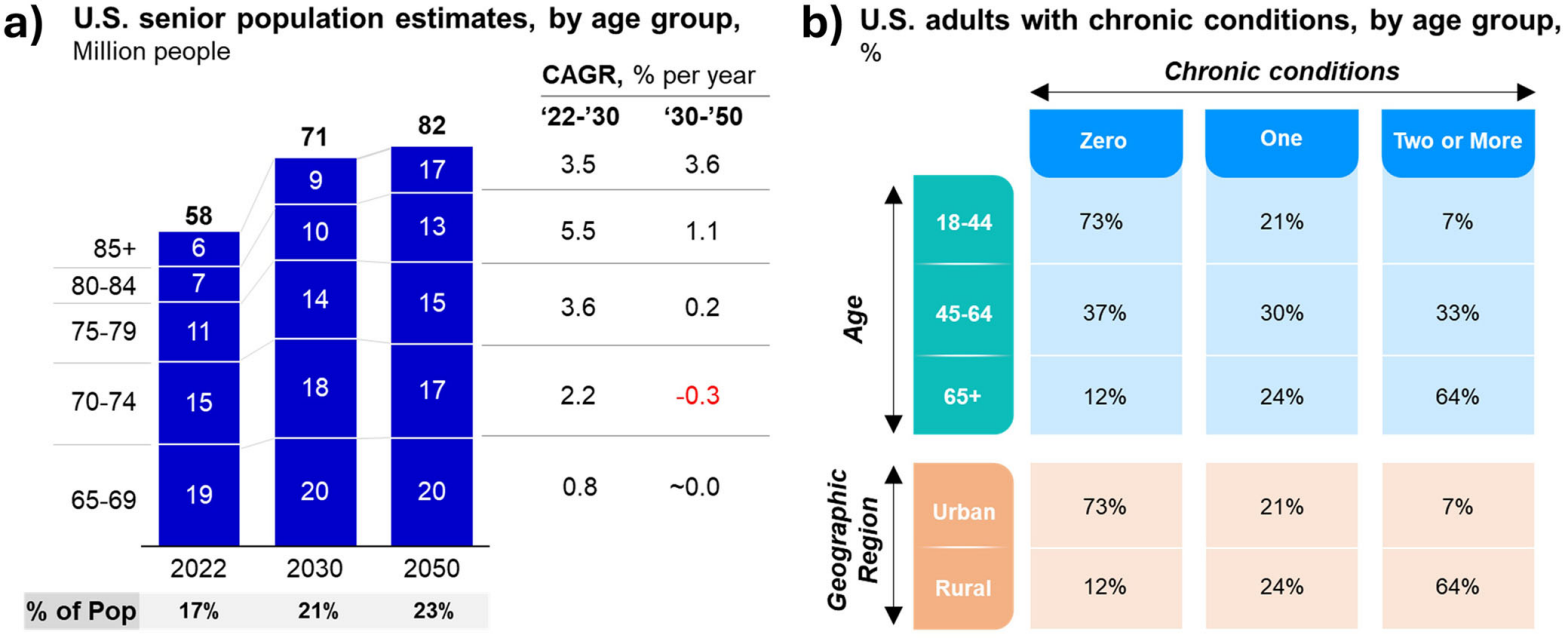
Growth of senior population and chronic disease burden. This figure illustrates the expected growth of the senior population and the associated increase in chronic disease burden. Raw data sourced from “2023 National Population Projections Tables: Main Series”, which utilizes official estimates of resident population on July 1, 2022 as the base for projecting the U.S. population from 2023 to 2100 (panel **a**) and Boersma, et al., “Prevalence of Multiple Chronic Conditions Among U.S. Adults, 2018” (panel **b**).

The U.S. population is aging rapidly because of two interrelated factors: the aging of the baby boomer generation, and the increase in life expectancy. The baby boomer generation constitutes the largest cohort in the U.S. history, with about 73 million members^11^. As this cohort reaches retirement age, the share of the population that is 65 and older will increase significantly, from 17% in 2022 to 21% in 2030, and to 23% in 2050 (Fig. 1A)^12^. By 2050, the number of Americans aged 65 and older will increase by 40%, from 58 million in 2022 to 82 million in 2050.

The aging population, particularly those over 85 years old, presents new challenges for the medical system. This will be the fastest growing segment, tripling in size from 6.5 million in 2022 to 17.3 million in 2050, a number comparable to the current population of New York state^13^. These elderly adults often suffer from multiple and complex health conditions, including age-related diseases that affect their heart, brain, and immune system. However, the medical system lacks the experience and expertise to effectively treat these diseases and provide specialized, personalized care for this vulnerable group. The increase in the share and size of the older population will have implications for the demand and supply of healthcare and social services, as well as for the economic and fiscal stability of the nation.

One of the main drivers of the increased healthcare demand and utilization among the elderly is the high prevalence of multiple chronic conditions (MCCs), which are defined as having two or more chronic diseases that last at least a year and require ongoing medical attention or limit activities of daily living^14,15^. According to the Centers for Disease Control and Prevention (CDC), 88% of older adults have at least one MCC, and 60% have at least two (Fig. 1B). These include common conditions such as hypertension, arthritis, heart disease, cancer, diabetes, and chronic kidney disease. MCCs are associated with increased mortality, disability, functional decline, and reduced quality of life. Moreover, they pose significant challenges to healthcare provision and management, as they require complex and coordinated care across multiple settings and providers. A study by Machlin et al. (2019) found that among Medicare beneficiaries aged 65 and older, those with MCCs accounted for 94% of total healthcare expenditures in 2010, compared to 6% for those without MCCs^16^. The average annual expenditure per person was $21,342 for those with four or more MCCs, $13,272 for those with three MCCs, $9176 for those with two MCCs, and $5865 for those with one MCC. These figures contrast sharply with the $2025 spent for those without any MCC.

The high prevalence of MCCs among the elderly is expected to persist or even increase in the future, as it is closely linked to the increase in life expectancy. As people live longer, they are more likely to develop and accumulate chronic diseases over time, especially if they have risk factors such as age-related physiological changes, environmental exposures, lifestyle behaviors, genetic predispositions, and social determinants of health. For example, a study by Crimmins and Beltrán-Sánchez^17^ found that the increase in life expectancy in the U.S. between 1998 and 2008 was accompanied by an increase in the number of years spent with MCCs, especially among the elderly^17^. The study estimated that the average number of years spent with MCCs increased from 7.2 to 8.6 for men aged 65 and older, and from 10.0 to 11.3 for women aged 65 and older. Therefore, the aging population will face a higher burden of chronic diseases and a lower quality of life in the coming decades. Making matters worse, the health and longevity of the next wave of aging people may also be affected by new external triggers, such as obesity, processed food intake, microbiome changes, climate change, pandemics, and pollution, which can have diverse and unpredictable impacts on different individuals. These triggers can also change the health behaviors and healthcare access of the elderly.

Another challenge that arises from the medication requirements of the aging population is polypharmacy, which is defined as the concurrent use of five or more medications^18^. Individuals aged 65 and over account for over a third of all prescribed medications in the U.S.^19^. However, polypharmacy can have negative consequences, such as increased risk of drug interactions, adverse drug events, medication non-adherence, and medication errors. These can lead to poor outcomes, such as reduced effectiveness, increased morbidity and mortality, and decreased quality of life. Therefore, polypharmacy necessitates careful medication management and monitoring, as well as regular medication reviews and deprescribing when appropriate. A study by Qato et al. ^20^ found that among U.S. adults aged 65 and older, the prevalence of polypharmacy increased from 31.4% in 1999-2000 to 35.8% in 2011-2012, and the prevalence of potentially inappropriate medication use increased from 8.7% to 10.0%^20^. The study also found that polypharmacy was associated with higher rates of emergency department visits and hospitalizations^20^.

The economic implications of the aging population for the healthcare sector are profound, as they affect not only the healthcare spending and resource utilization, but also the healthcare workforce, the healthcare quality, and the healthcare innovation. According to the Congressional Budget Office, the federal spending on major health programs for the elderly, such as Medicare and Medicaid, will increase from 6.6% of gross domestic product (GDP) in 2020 to 9.2% of GDP in 2050^21^. This projected growth is presumably driven by the older age segments differing healthcare utilization patterns and the increasing complexity of care. Specifically, it is estimated that adults aged 65+ visits doctors 20% more frequently than younger adults and experience a threefold increase in hospitalization rates^22^. This increased utilization and complexity of care drives increased spending as reported in a 2019 Kaiser Family Foundation study that found Medicare spending for beneficiaries aged 65 to 74 averages $7566, which nearly doubles to $16,145 for those aged 85 and older^23^.

The rising tide of healthcare needs due to an aging population is multifaceted, encompassing increased service utilization, higher prevalence of chronic diseases, escalated healthcare spending, and complex medication management. This scenario places unprecedented demands on the healthcare system, calling for innovative approaches in care delivery, financial planning, and resource allocation. Adapting to these changes requires a concerted effort from healthcare providers, policymakers, and stakeholders to ensure that the system is not only responsive but also sustainable in meeting the evolving needs of an aging society. Therefore, it is imperative to recognize and discuss the impact this evolution will have on the demand for healthcare professionals, such as physicians, nurses, pharmacists, and specialists in geriatrics, who will be the critical in providing adequate and appropriate care for the elderly with complex medical needs.

## The overburdened healthcare landscape: healthcare delivery challenges

The demand for healthcare workers is expected to outpace the supply, resulting in a projected deficit of 1.2 million registered nurses and 121,900 physicians by 2030^8,21^. The healthcare workforce shortage is driven by several factors, such as the aging of the workforce itself, the insufficient supply of new entrants, the uneven distribution across regions and specialties, and the increased workload and stress of the workers. These factors are summarized in Table 1, along with their implications and notable statistics.

**Table 1:** Challenges and impacts of workforce dynamics in healthcare

Factor	Implication	Notable Statistics
The aging of the healthcare workforce itself, leading to increased retirements and reduced working hours.	• The system will face a significant loss of experience and expertise, as well as a reduced availability of workers.	• According to the Health Resources and Services Administration, about a third of the current registered nurses are over 50 years old, and about half of the current physicians are over 55 years old^82,83^.
The insufficient supply of new entrants into the healthcare professions, due to limited educational capacity, high attrition rates, and low retention rates.	• The system will face a shortage of qualified and skilled workers, as well as a lack of diversity and representation in the workforce. • The supply of new healthcare graduates will continue to be constrained by the limited capacity of educational institutions, the high costs and debts of education, and the competitive and demanding nature of the professions^82–84^.	• According to the American Association of Colleges of Nursing, U.S. nursing schools turned away more than 75,000 qualified applicants in 2018 due to faculty shortages, insufficient clinical sites, and budget constraints^85^. • According to the AAMC, U.S. medical schools have increased their enrollment by 31% since 2002, but this is still not enough to meet the projected demand for physicians^85^.
The uneven distribution of the healthcare workforce across geographic regions, specialties, and settings, resulting in shortages in rural and remote areas, primary care and geriatric care, and community-based and home-based care.	• The system will face disparities and gaps in access and quality of care for different populations, especially the elderly, who often have multiple and complex needs. • The distribution of healthcare workers will become even more skewed towards urban and affluent areas, leaving rural and remote areas with fewer and less accessible providers^83,84^.	• According to the Health Resources and Services Administration, about 60 million Americans live in areas with a shortage of primary care providers, and about 77 million Americans live in areas with a shortage of mental health providers^86^. • According to the American Geriatrics Society, there are only about 7,300 certified geriatricians in the U.S., which is far below the estimated need of 30,000 by 2030^87^.
The increased workload and stress of the healthcare workers, leading to burnout, dissatisfaction, and turnover	• Attrition of healthcare provider could increase leading to overall reduction in available staff.	• According to a survey by the American Nurses Association, more than half of the nurses reported feeling overwhelmed by their work, and more than a third reported feeling emotionally exhausted^88^. • According to a survey by the Medscape, more than 40% of the physicians reported feeling burned out, and more than 10% reported feeling depressed^89^.

The shortage of healthcare providers will have a ripple effect on the entire healthcare system, affecting the quality, accessibility, and affordability of care. Physician shortages lead to increased mortality, reduced preventive care, and higher healthcare spending^24^. Moreover, the shortage of physicians creates a competitive environment for talent, where healthcare providers vie for the limited pool of available professionals. This competition may result in sector consolidation, where larger and more affluent providers acquire or merge with smaller and less profitable ones, creating economies of scale and scope. However, this consolidation may also have negative consequences, such as reduced competition, increased market power, and higher prices^25^.

Appropriate staffing and labor supply are necessary for delivering care, but they are not sufficient without adequate resources and infrastructure. However, the current system is not well prepared to handle the increase in volume and complexity of care, resulting in overcrowding, wait times, delays, cancellations, and rationing of care. Some of the factors that contribute to this resource gap include:

- The state and performance of care delivery. The U.S. infrastructure is in bad shape and needs more investment and improvement, as a 2017 report by the American Society of Civil Engineers gave it a D+ grade^26^. The system wastes about $750 billion, or 30% of its spending, every year on unnecessary or excessive costs, fraud, and other inefficiencies^27^. It also has high variation in the quality and results of care across different providers, places, and regions, which can lead to too much, too little, or improper use of services^28^. For instance, a report by the Dartmouth Atlas Project showed that Medicare spending per beneficiary ranged by more than three times across regions, and that more spending did not mean better quality or satisfaction^29^.
- The lack and imbalance of beds. The U.S. has seen a decline in the number of hospital beds per person from 4.5 in 1980 to 2.4 in 2018, reflecting the move from inpatient to outpatient care and the attempts to save costs and enhance efficiency. However, this trend also implies that there is less excess capacity to cope with fluctuations in demand, such as during pandemics, disasters, or seasonal variations. Furthermore, the allocation of beds across states and regions is unequal, creating differences in access and quality of care for various populations^30^. For example, the states with the lowest number of beds per person are Nevada (1.8), Oregon (1.8), and Washington (1.9), while the states with the highest number of beds per person are South Dakota (4.1), North Dakota (4.0), and West Virginia (3.8)^31^.
- The inadequacy and inefficiency of technology. The U.S. healthcare system is lagging behind in the adoption and use of information and communication technology (ICT), such as electronic health records (EHRs), telemedicine, and health information exchange (HIE), which can improve the quality, safety, and coordination of care, as well as reduce the costs and errors of care^32^. According to a 2023 report by the Organisation for Economic Co-operation and Development (OECD), the U.S. only hit the threshold of EHR use in 90% of physician offices, medical specialist offices, hospitals, and emergency rooms in 2021^33^. Although the U.S. joins 17-21 other countries that, depending on the setting, have achieved this milestone, it is one of the four countries that did not report having a mandated system in place^33^. As a result, access to records is inconsistent and may require use of multiple portals to view all of a patient’s medical data, impeding benefits that may be observed through shared medial data across practices^33^. For example, analysis of EHRs have supported efforts to predict risk of conditions such as gestational diabetes^34^ and postpartum depression^35^ as well as to evaluate medical trends during the COVID-19 pandemic^36–38^. The large volume of data used in such efforts has generated interest for the application of machine-learning, particularly deep learning, to parse through complex and multivariate relationships identifiable within patient records^39–41^. Despite the potential, there are various concerns that arise with the digitization and availability of such records, such as breaches through cyber-attacks^42^. Finding avenues to address such concerns regarding patient privacy will be an important step towards realizing the benefits from advances in EHRs and their analysis to identify health trends.

The resource gap in the U.S. healthcare system will have serious consequences for the health and well-being of the population, especially the elderly, who are more vulnerable and dependent on the availability and quality of care. A study by the Commonwealth Fund found that the U.S. ranked last among 11 high-income countries in the health outcomes and experiences of older adults, with the highest rates of mortality, disability, hospitalizations, and unmet needs^43^. Moreover, the resource gap will have implications for the innovation and competitiveness of the U.S. healthcare sector, as it will limit the ability and opportunity to develop and implement new and better ways of delivering and improving care, such as digital health, precision medicine, and artificial intelligence (AI)^44^.

The U.S. healthcare system is facing a supply crisis, as it is unable to meet the rising and complex needs of the aging population. The system is suffering from a shortage of labor and a constraint of resources, resulting in a capacity gap that affects the efficiency, equity, and quality of care. Addressing this crisis requires a strategic and comprehensive approach that involves increasing the quantity and quality of the healthcare workforce, enhancing the availability and accessibility of the healthcare resources, and improving the performance and productivity of the healthcare delivery. Achieving these goals requires collaboration and coordination among the healthcare providers, policymakers, and stakeholders, as well as a commitment and investment in the healthcare sector.

## The fragmentation and disparity in healthcare provision: access challenges and the “rich-poor divide”

The U.S. healthcare system is facing a challenge not only in meeting the demand and supply of healthcare, but also in ensuring that the healthcare is accessible and affordable for all segments of the population, especially the elderly, who often face barriers and difficulties in obtaining and utilizing the care they need. The system is characterized by fragmentation and disparity, meaning that the healthcare provision is divided and disconnected across different providers, payers, and settings, and that the healthcare outcomes and experiences vary widely across different groups, regions, and conditions. These features of the system create inefficiencies, inequities, and inconsistencies in the access and quality of care, which can have negative impacts on the health and well-being of the population.

The fragmentation of the U.S. healthcare system stems from the lack of a universal and integrated system of healthcare coverage and delivery, which leads to gaps and overlaps in the coordination, continuity, and comprehensiveness of care^45^. The system is composed of multiple and competing payers, such as private insurers, public programs, and self-pay individuals, each with their own eligibility criteria, benefit packages, payment mechanisms, and administrative rules. This creates a complex and confusing landscape for the consumers and the providers, who have to navigate through different and often conflicting policies, procedures, and requirements. Moreover, the system is composed of multiple and independent providers, such as hospitals, clinics, physicians, nurses, pharmacists, and others, each with their own practice patterns, quality standards, and information systems. This creates a siloed and disjointed landscape for the delivery and management of care, which can result in duplication, fragmentation, and gaps in the care process.

The fragmentation of the U.S. healthcare system has significant implications for the access and quality of care, especially for the elderly, who often have multiple and complex needs that require coordinated and comprehensive care across different settings and providers. The fragmentation can lead to poor outcomes, such as increased errors, complications, readmissions, and costs, as well as reduced satisfaction, trust, and adherence^46^. For example, a study by Pham et al.^47^ found that among Medicare beneficiaries aged 65 and older, those who had four or more chronic conditions and saw 10 or more physicians had twice the rate of preventable hospitalizations than those who saw two or fewer physicians^47^. Moreover, the fragmentation can lead to unmet needs, such as delayed or foregone care, as well as increased burden, such as out-of-pocket expenses, transportation difficulties, and caregiving responsibilities^48^. For example, among Medicare beneficiaries aged 65 and older, 15% reported having trouble getting timely appointments, 12% reported having trouble getting needed tests or treatments, and 9% reported having trouble getting needed medications^49^.

The disparity in the U.S. healthcare system stems from the unequal and unfair distribution of healthcare resources, opportunities, and outcomes across different groups, regions, and conditions, which leads to gaps and differences in the access, quality, and affordability of care^50^. The system is influenced by various factors, such as income, education, race, ethnicity, gender, age, geography, and disability, that affect the health status and health behaviors of the population, as well as the availability and utilization of healthcare services. These factors create a diverse and heterogeneous landscape for the consumers and the providers, who face different and often disproportionate challenges and barriers in obtaining and delivering care. Moreover, the system is influenced by various policies, programs, and practices, such as reimbursement rates, quality measures, and incentives, that affect the allocation and distribution of healthcare resources, such as workforce, facilities, equipment, and technology. These policies, programs, and practices create a dynamic and complex landscape for the payers and the policymakers, who must balance and align the competing and conflicting interests and objectives of the healthcare stakeholders.

As the demand for healthcare services increases due to the aging population, and the supply of healthcare workers and resources remains insufficient and inadequate, a new form of fragmentation and disparity is emerging in the U.S. healthcare system: the rich-poor divide. This refers to the phenomenon where the affluent and urban areas attract and retain more and better healthcare professionals and facilities, while the poor and rural areas are left with fewer and worse healthcare options. This creates a vicious cycle, where the rich areas have more access and quality of care, and the poor areas have less access and quality of care, leading to further widening of the health and economic gaps between them.

One of the factors that contributes to this new form of fragmentation and disparity is the market-driven and competitive nature of the U.S. healthcare sector, where healthcare providers are motivated by financial incentives and rewards to work in areas and specialties that offer higher compensation and recognition. This creates a situation where the supply of healthcare workers is skewed towards the areas and specialties that have more demand and resources, such as urban and affluent areas, and specialty and subspecialty care. Conversely, the supply of healthcare workers is scarce in the areas and specialties that have less demand and resources, such as rural and remote areas, and primary and geriatric care. This results in a mismatch between the needs and the availability of the healthcare workforce, which affects the access and quality of care for different populations.

One of the examples that illustrates this new form of fragmentation and disparity is the rise of travel nurses, who are registered nurses with advanced training and certification in various specialties, and work on a temporary or contract basis in different locations and settings. These nurses are in high demand, as they can fill the gaps and shortages of anesthesiologists (i.e., travel nurse anesthetist) or other nurse specialists, who are often concentrated in urban and academic centers. Moreover, these nurses are well compensated, as they can earn significantly higher salaries and benefits than regular nurses and have more flexibility and autonomy in choosing their assignments and schedules. However, these nurses also contribute to the fragmentation and disparity of the healthcare system, as they tend to work in areas and settings that offer more opportunities and rewards, such as affluent and urban areas, and private and specialty hospitals. This leaves the areas and settings that have less opportunities and rewards, such as poor and rural areas, and public and primary care facilities, with fewer and less qualified healthcare workers, which affects the access and quality of care for the populations they serve.

Addressing the challenge of fragmentation and disparity in the U.S. healthcare system requires a holistic and integrated approach that involves improving the coordination and continuity of care, enhancing the equity and inclusivity of care, and ensuring the affordability and sustainability of care. Achieving these goals requires collaboration and coordination among the healthcare providers, payers, policymakers, and stakeholders, as well as a commitment and investment in the healthcare sector.

## Interventions, policy reform, and global comparison

As the number of individuals living into their 80s, 90s and beyond increases dramatically, focus has shifted from extending lifespan to enhancing the quality of these additional years. This approach, known as ‘delayed aging,’ has encompassed investments in technology and policies that increase the number of years lived without the accumulation of chronic conditions and other side effects of aging. Achieving this would result in the compression of morbidity, meaning that chronic illnesses would be concentrated into a shorter period towards the end of life. This shift would not only have an impact on the general health of the population, would also have a positive financial impact. For example, a 2013 study conducted by Goldman et al. estimated that wide-spread delayed aging would save the U.S. $7.1 trillion by 2060.

The emerging field of geroscience is central to increasing the number of healthy years in older populations. This discipline seeks to understand the relationship between aging and age-related diseases, aiming to mitigate the latter by targeting the biological processes of aging itself^51^. A key strategy involves identifying biomarkers and risk factors, such as socioeconomic and lifestyle choices, that predict disease development in later life (Table 2). Technological advancements have enabled the aggregation of large multi-Omics datasets and longitudinal medical records from diverse patient groups and different aging tissues^52^. These include blood, brain, muscle, heart, liver, joint, skeleton, fat, among others. For example, the Accelerating Medicines Partnership^®^’s (AMP^®^’s) Alzheimer’s Disease program has used a multi-omic analyses of molecular data from human brain samples to identify over 500 unique drug candidate targets^53^. This wealth of information offers an unprecedented opportunity to leverage AI methodologies to decipher unique patient markers and identify potential interventions.

**Table 2:** Diagnostic biomarkers for age-related diseases and their implications for clinical practice

Diagnostic Risk Factor(s)	Select Biomarkers	Change with Age	Physiological Impact	Associated Diseases	Testing	Use in Medicine	Ref
Chronic Inflamm. (Inflamm-ageing)	CRP; IL-6; TNF-α; IL-1β	Increase	Leads to system inflamm. causing tissue damage, altered immune response, and increase risk of ASCVD.	CV diseases; Frailty; Dementia; CKD; DM; Cancer; Depression; Sacropenia	Blood tests; High Sensitivity CRP Test	Utilized currently	^90^
Metabolic Health (Glucose Metabolism, Hyper-glycemia)	Fasting glucose; HbA1c; Insulin levels	Dysreg.	Impairs glucose utilization, leading to hyperglycemia and contributing to vascular damage and insulin resistance.	CV disease; Cognitive decline; T2D; Metabolic syndrome	Blood tests (HbA1c, glucose testing, insulin assay)	Utilized currently	^91^
Muscle Function (Sarcopenia)	Muscle mass; Grip strength; Gait speed; DEXA	Decrease	Reduces skeletal muscle strength and function, impairs mobility and increases fall risk	Sarcopenia; Frailty; Increased risk of falls and hospitalization; Decreased mobility and independence	Physical assessments; DXA; CT; MRI; BIA	Utilized currently	^92^
Cachexia/Obesity	BMI; Waist CIR; Body comp.	Varies	Alters metabolic regulation, increases mechanical load on joints, and contributes to systemic inflamm.	Diabetes; CV diseases; Osteoarthritis; Sleep apnea; Certain cancers (e.g., breast, colon); Surgical Risks and complications	Physical measurement; DXA; BIA	Utilized currently	^93^
Immune Aging Fingerprint	Immuno-phenotyping; Senescent cell markers; CD28null T cells	Immune Dysreg.	Leads to decreased immune surveillance and increased prevalence of senescent cells contributing to tissue dysfunction.	Increased susceptibility to infections; Autoimmune disorders; Cancer; Reduced VE	Flow cytometry; SA-β-gal staining; Immuno-phenotyping	Soon to be diagnostic	^94^
Genetic Risk Factors	Genetic poly-morphisms; SNPs	Stable but influence disease risk	Influences metabolic pathways and immune responses, predisposing individuals to various chronic conditions.	Metabolic syndrome; CV diseases; Alzheimer’s disease; Certain cancers; Response to medications (pharmaco-genomics)	Genetic screening; SNP arrays	Exploratory	^95,96^
Microbiome	Microbial comp.; SCFA profiles	Changes with diet, antibiotic usage	Affects gut barrier function, systemic inflamm., and nutrient metabolism.	GI disorders; Metabolic dysregulation (e.g., obesity, diabetes); Autoimmune diseases; Mood disorders	16 S rRNA gene sequencing; Metagenomic sequencing	Exploratory	^90^
Telomere Length	Telomere length	Shortens	Shortening of telomeres is associated with cellular aging and increased risk of age-related diseases.	CV diseases, various cancers; OP; Diabetes; Increased risk of mortality	Quantitative PCR; Telomere length analysis	Exploratory	^97^
Oxidative Stress Markers	MDA; 8-OHdG; Antioxidant capacity	Increases	Causes cellular damage and contributes to the aging process and development of age-related diseases.	NDDs; CV diseases; Cancer; AMD	Blood tests; ELISA; Spectro-photometry	Exploratory	^98^
Vitamin D Levels	25-hydroxy-vitamin D	Varies with exposure and intake	Influences bone health, immune function, and has been linked to a lower risk of several chronic diseases.	OP; CV diseases; T2D; MS; Depression; Certain Cancers	Blood tests (25-hydroxy-vitamin D assay)	Exploratory	^99^

*8-OHdG* 8-hydroxy-2′-deoxyguanosine, *AMD* age-related macular degeneration, *ASCVD* atherosclerotic cardiovascular disease, *BIA* bioelectrical impedance analysis, *BMI* body mass index, *CIR* circumference, *CKD* chronic kidney disease, *comp.* composition, *CPR* C-reactive protein, *CT* computed tomography, *CV* cardiovascular disease, *DEXA* dual-energy x-ray absorptiometry, *DM* diabetes mellitus, *Dysreg.* dysregulation, *ELISA* enzyme-linked immunosorbent assay, *GI* gastrointestinal disease, *HbA1c* hemoglobin A1C, *IL* interleukin, *MDA* malondialdehyde, *MRI* magnetic resonance imaging, *MS* multiple sclerosis, *NDDs* neurodegenerative diseases, *OP* osteoporosis, *PCR* polymerase chain reaction, *Ref* references, *SA-β-gal* senescence-associated β-galactosidase, *SCFA* short chain fatty acids, *SNPs* single nucleotide polymorphisms, *T2D* Type 2 diabetes, *TNF-α* tumor necrosis factor-α, *VE* vaccine efficacy.

Once these risk factors are identified, researchers can develop interventions that correct or mitigate them. These include addressing issues such as immune aging, chronic low-grade tissue inflammation, obesity, mitochondrial age-related insufficiency, and brain proteinopathies^54^. For instance, clinical studies with new targeted immune agents aim to rejuvenate the aging immune system using immune aging biomarkers as surrogate endpoints^55^. The hope is that restoring immune health could translate to beneficial downstream effects on the vascular, heart, brain, and kidney systems. The advent of the GLP-1 drug class (e.g., Semaglutide and Tirzepatide), which not only have the potential to prevent obesity-related diseases later in life, also offers a promising avenue for the reversal of metabolic aging and may even promote DNA repair in neurodegenerative diseases^56^. Maintaining muscle function is also crucial, particularly in the context of aging or chronic conditions like sarcopenia^57^. Insights into muscle augmenting factors and the ability to mobilize and differentiate muscle stem cells present critical areas for promoting healthy aging^58^. Bioengineering, including the development of exoskeletons for spinal injury patients, offers potential solutions for maintaining ambulation during aging^59^.

Future interventions may include longitudinal analyses of genome integrity and maintaining DNA fidelity systems. Reducing the accumulation of somatic mutations, which correlate with aging of bone marrow^60^, myeloid cell dysplasia (clonal hematopoiesis)^61^, vascular wall dysfunction^62^, and blood cancers^63^, could be possible. The first successful trials in gene therapy and gene editing have shed new light and promise on human disease, suggesting that restoring the integrity of the human genome and cell systems may increasingly be within our technological reach. This could expand to restore function in autoimmune and fibrotic diseases, further underscoring the transformative potential of geroscience and technology in improving healthcare for the elderly.

As the field of geroscience advances, there is a significant risk that these innovations may predominantly benefit those with substantial resources, further exacerbating the ‘rich-poor’ divide in eldercare and highlighting one type of inequity that tomorrow’s elderly may face. There is already evidence that compression of morbidity may be due more to socioeconomic factors than biological determinants^64,65^. However, we do not yet know what other challenges these future generations may face. New and emerging socioeconomic factors and factors associated with marginalized groups in younger populations will have a yet unknown impact on tomorrow’s elderly. The shifting healthcare requirements of younger demographics, characterized by factors such as the increase in gender-affirming care, escalating mental health issues, and substance misuse, including opioids, are likely to affect the future landscape of elderly care in ways that are currently unknown. The opioid crisis, as elaborated in Barbara Kingsolver’s *Demon Copperhead*, serves as an illustration of how prevalent health and societal challenges can have significant long-term impacts on public health and healthcare systems^66^. This narrative, which mirrors the broader societal problem of substance abuse, underscores the importance of incorporating equity as a fundamental factor in healthcare policy decisions. Ensuring that healthcare improvements, including those emerging from geroscience, are accessible to all, irrespective of their socioeconomic status, is vital to prevent further exacerbation of the economic disparity in eldercare.

To this end, the U.S. has launched policy reforms over the last two decades aimed at securing equitable healthcare for its senior citizens. These measures seek to confront salient challenges in the accessibility, quality, and financial viability of services catering to the elderly. The expansion of Medicaid under the Affordable Care Act (ACA), enacted in 2010, serves as a cornerstone of these initiatives, enhancing Medicaid’s scope to encompass superior benefits, cost reductions, and improved eldercare^67^. Significantly, the ACA reducing the Medicare Part D prescription drug “donut hole,” or drug coverage gaps, improving preventive care with no cost-sharing. Additional initiatives, such as Medicare Advantage, aim to improve eldercare coordination by allowing Medicare beneficiaries to enroll in managed care plans (Medicare Advantage). Medicare Advantage, for example, enrolled over 30.8 million individuals in 2023 alone, and now represent over 50% of all seniors on Medicare^68^.

As components of the patient protection models, accountable care organizations (ACOs) have also exhibited efficacy in enhancing care coordination, leading to improved patient outcomes and decreased costs. A 2021 report from the Centers for Medicare & Medicaid Services (CMS) asserts that ACOs yielded $17.7 billion in gross savings and $6.5 billion in net savings for Medicare between 2021 and 2022, while also improving the quality of care^69^. Alongside these models, enhancements in Long-Term Care and Support Services through the expansion of home and community-based services (HCBS) via Medicaid have substantially widened access to personalized long-term care^70,71^. These services are aimed at facilitating elderly individuals to ‘age in place’^72^. In 2019 alone, Medicaid HCBS expenditures amounted to $162 billion, highlighting a sustained trend towards increased investment in services that promote autonomy and dignity for the elderly^73^. Additionally, this represents a cost-effective approach to long-term care by minimizing dependence on more costly institutional care settings.

Internationally, countries have adopted diverse strategies to address the healthcare needs of their aging populations. Japan and Germany, for example, both emphasize integrated care models and insurance-based solutions, like Germany’s statutory health insurance complemented by long-term care insurance^74,75^ and Japan’s Long-Term Care Insurance^76^. However, Japan, as one of the world’s oldest populations, faces unique challenges in sustaining its healthcare workforce^77^. In an attempt to compensate for this, this country has positioned itself at the forefront of automating elder care including the development of technologies such as ‘care robots,’ to improve the quality of care in nursing homes^78^. Similar to Japan, Singapore has also implemented technological healthcare solutions for its aging population, investing in improving telemedicine, remote health monitoring apps, wearable wellness technology, and smart home solutions (e.g., fall detect sensors) to facilitate independent living and reduce the burden on the healthcare system^79^. This country is currently second in the word in terms of highest life expectancy, compared with the U.S. at 48^80^. Sweden, number 20 on that list^80^, is internationally recognized as a model for eldercare. It is another country that heavily focuses on ‘aging in place’ by providing heavily funded care through municipal taxes and government grants^81^.

## Conclusion

It is often said that there is always tomorrow, implying that we can postpone our actions and decisions to a later date. However, this sentiment cannot hold true anymore when it comes to the care of the aging population. For us to have a prosperous tomorrow, we must begin planning and actioning today. The aging of the population is not a distant or hypothetical scenario, but a present and inevitable reality. We cannot afford to wait and see what happens. We must act and change what happens today. We have the potential and the responsibility to create a better and brighter future for the elderly and for ourselves. The question is: will we?

## Data Availability

No datasets were generated or analyzed for this manuscript.
