# Author Correction: Healthcare on the brink: navigating the challenges of an aging society in the United States

**DOI:** 10.1038/s41514-024-00153-5

**Published:** 2024-05-10

**Authors:** Charles H. Jones, Mikael Dolsten

**Affiliations:** Pfizer, 66 Hudson Boulevard, New York, New York, 10018 USA

**Keywords:** Geriatrics, Health services, Public health, Epidemiology

Correction to: *npj Aging* 10.1038/s41514-024-00148-2, published online 06 April 2024

In this article in section “The overburdened healthcare landscape: healthcare delivery challenges”, in the first bullet list ‘The U.S. healthcare infrastructure is in bad shape and needs more investment and improvement, as a 2017 report by the American Society of Civil Engineers gave it a D+ grade.” the word ‘healthcare’ was included inadvertently. This word has now been removed from the HTML and PDF versions of the article.

